# ArUco‐based stylus reliability for reproductible 3D digitalisation of shoulder cartilage contours

**DOI:** 10.1111/joa.13987

**Published:** 2024-01-12

**Authors:** Florent Moissenet, Christian Elmo Kulanesan, Kevin Co, Pablo Rodriguez, Pierre Vacher, Jean‐Yves Beaulieu, Nicolas Holzer

**Affiliations:** ^1^ Biomechanics Laboratory Geneva University Hospitals and University of Geneva Geneva Switzerland; ^2^ Kinesiology Laboratory Geneva University Hospitals and University of Geneva Geneva Switzerland; ^3^ Université Savoie Mont Blanc Laboratoire SYMME Annecy‐le‐Vieux France; ^4^ Laboratory of Simulation and Movement Modeling, School of Kinesiology and Exercise Sciences Université de Montréal Montreal Quebec Canada; ^5^ Department of Surgery Geneva University Hospitals Geneva Switzerland; ^6^ Department of Anatomy, Faculty of Medicine University of Geneva Geneva Switzerland

**Keywords:** 3D digitalisation, ArUco markers, cartilage, hand‐held stylus, shoulder

## Abstract

Imaging techniques in anatomy have developed rapidly over the last decades through the emergence of various 3D scanning systems. Depending on the dissection level, non‐contact or tactile contact methods can be applied on the targeted structure. The aim of this study was to assess the inter and intra‐observer reproducibility of an ArUco‐based localisation stylus, that is, a manual technique on a hand‐held stylus. Ten fresh–frozen, unembalmed adult arms were used to digitalise the glenoid cartilage related to the glenohumeral joint and the contour of the clavicle cartilage related to the acromioclavicular joint. Three operators performed consecutive digitalisations of each cartilage contour using an ArUco‐based localisation stylus recorded by a single monocular camera. The shape of each cartilage was defined by nine shape parameters. Intra‐observer repeatability and inter‐observer reproducibility were computed using an intra‐class correlation (ICC) for each of these parameters. Overall, 35.2 ± 2.4 s and 26.6 ± 10.2 s were required by each examiner to digitalise the contour of a glenoid and acromioclavicular cartilage, respectively. For most parameters, good‐to‐excellent agreements were observed concerning intra‐observer (ICC ranging between 0.81 and 1.00) and inter‐observer (ICC ranging between 0.75 and 0.99) reproducibility. To conclude, through a fast and versatile process, the use of an ArUco‐based localisation stylus can be a reliable low‐cost alternative to conventional imaging methods to digitalise shoulder cartilage contours.

## Graphical abstract

The aim of this study was to assess the inter and intra‐observer reproducibility of an ArUco‐based localisation stylus to digitalise the glenoid cartilage related to the glenohumeral joint and the contour of the clavicle cartilage related to the acromioclavicular joint. For most parameters, good‐to‐excellent agreements were observed concerning intra‐observer (intra‐class correlation [ICC] ranging between 0.81 and 1.00) and inter‐observer (ICC ranging between 0.75 and 0.99) reproducibility.

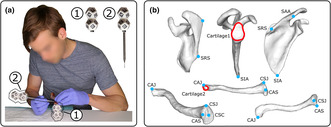

## 1 INTRODUCTION

Imaging techniques in anatomy have developed rapidly over the last decades through the emergence of various 3D scanning systems (Sindhu & Soundarapandian, 2019). Depending on the dissection level, non‐contact or tactile contact methods can be applied on the targeted structure (Sindhu & Soundarapandian, 2019). On one hand, non‐contact methods are widely represented by medical imaging techniques (e.g., CT, MRI, x‐rays, and ultrasound) (Haleem & Javaid, 2019), as well as surface scanning techniques (e.g., time of flight, laser, and structured lighting) (Sood et al., 2021). On the other hand, tactile contact methods can be based on automatic techniques such as coordinate measuring machine and robotic arms (Sindhu & Soundarapandian, 2019) or manual techniques often based on a hand‐held stylus (Hargett et al., 2020). This last category offers a versatile approach to scan structures using any tracking system and allows 3D scanning at reduced cost.

Recent developments in vision introduced new approaches for optical tracking based on binary square fiducial markers. Garrido‐Jurado et al. (2014) suggested highly reliable fiducial markers called ArUco markers. On this basis, several authors proposed to combine multiple ArUco markers to define a hand‐held stylus (Le et al., 2022). Their studies demonstrated that this approach could lead to a sub‐millimetric accuracy (0.4 mm at 60 Hz over a 30 × 40 cm with a 2.1 Mpx camera) working area tracking with a low‐cost system based on a simple 2D camera based on monocular vision. Still, to the best of our knowledge, the reliability of an ArUco‐based localisation stylus remains unknown. Such an assessment is yet required to estimate the measurement error of this instrument and its reproducibility across examiners.

The aim of this study was to assess the inter and intra‐observer reproducibility of an ArUco‐based localisation stylus. This was tested during the 3D digitalisation of shoulder cartilage contours. To do so, three operators digitalised the contour of the glenoid cartilage related to the glenohumeral joint and the contour of the clavicle cartilage related to the acromioclavicular joint. It was hypothesised that the use of the ArUco‐based localisation stylus allows for the 3D digitalisation of cartilage contours, or other anatomical structures, in a simple, versatile, and reliable process.

## 2 METHODS

### 2.1 Specimen preparation

The sample size required for this study was computed using the *ICC.Sample.Size* R package (v. 1.0) (R‐Core‐Team, 2020a) (R 4.1.2 and RStudio version 2021.09.0 build 351) where intra‐class correlation (ICC) was defined as the primary outcome (see Section 2.5). Based on previous experiments involving ex vivo knee ligament attachments digitalised with a hand‐held stylus (Athwal et al., 2020), the hypothesised ICC value was set to 0.9. The resulting requested sample size was *N* = 8. To anticipate potential dropouts, 10 fresh–frozen, unembalmed adult arms (five right sides and five left sides) were obtained from a previous study (Moissenet et al., 2022). None of the limbs had an advanced degenerative joint disease or previous ligamentous injury confirmed by direct inspection and radiographs before experiments. All pieces were acquired at the Anatomy Teaching Unit of the Geneva Faculty of Medicine. They were all selected from the local body donation programme. The Cantonal Commission for Research Ethics approved this study (2020‐00598). All procedures were performed in accordance with ethical standards of the institutional research committee and with the 1964 Helsinki Declaration and later amendments.

All pieces were stored at −20° and thawed at room temperature during approximately 72 h prior to testing. Dissection of the pieces consisted in resecting all the soft parts to preserve only bones and cartilage. An anatomical set of 10 dissected scapulas and clavicles was then used in this study.

### 2.2 Cartilage contour digitalisation

**FIGURE 1:**
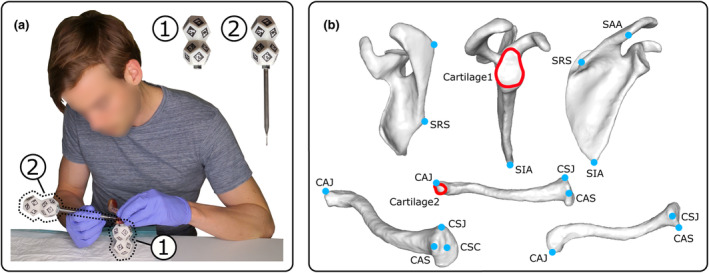
Cartilage contour digitalisation procedure. (a) Use of a stylus (2), equipped with ArUco markers on a double dodecahedron, to digitalise anatomical landmarks and cartilage contours. The bone is also instrumented with a double dodecahedron with ArUco markers (1). (b) Illustration of the digitalised anatomical landmarks and cartilage contours (see Table 1 for details). The nomenclature used for anatomical landmarks is based on the propositions made by Van Sint Jan (2007).

Cartilage contours were digitalised using an ArUco‐based localisation stylus (Figure 1). The 3D localisation of the stylus tip (1‐mm radius spherical contact probe) was made available using a set of planar ArUco markers (Garrido‐Jurado et al., 2014) glued on the surface of a double dodecahedron secured at an the other extremity of the stylus.

The position of ArUco markers was recorded by a 3.1‐Mpx monocular camera (EXO252 MU3, SVS‐Vistek, Germany) equipped with a 12‐mm focus camera zoom length (PENTAX TV, PENTAX, Japan) at each motion frame (10 Hz). After a preliminary calibration phase, the 3D position of each marker and of the stylus tip was stored in the stylus reference coordinate system, arbitrarily defined (Wu et al., 2017). A double dodecahedron was used in this study to avoid coplanarity (Schweighofer & Pinz, 2006). In such conditions, the stylus tip can be located irrespective of its position with a standard deviation of 0.3 mm along the camera optical axis and 0.1 mm along the two perpendicular axes (Elmo Kulanesan, 2023). For that, at least two ArUco markers must be visible by the camera, with sufficient depth of field and negligible motion blur.

The digitalisation process was performed sequentially. First, a set of anatomical landmarks (Figure 1; Table 1) were pointed by one examiner (PR) to define bone coordinate systems (see Section 2.3). Second, the contour of the glenoid cartilage related to the glenohumeral joint and the contour of the clavicle cartilage related to the acromioclavicular joint were digitalised. This choice was guided by temporal constraints and to assess cartilages with different sizes and shapes. To assess the inter and intra‐observer reliability of this digitalisation procedure, three examiners (PR, CE, and FM) performed three consecutive digitalisations of each cartilage contour.

**TABLE 1:** Digitalised anatomical landmarks and cartilage contours. The nomenclature used for anatomical landmarks is based on the propositions made by Van Sint Jan (2007).

Type	Bone	ID	Description
Anatomical landmarks	Scapula	SIA	Most distal point of the scapula lower angle
		SRS	Lateral point of the spine base triangle
		SAA	Acromial angle
	Clavicle	CAJ	Superior edge centre of the acromioclavicular joint on the clavicle
		CSJ	Superior edge centre of the sternoclavicular joint on the clavicle
		CAS	Anterior border apex of the sternoclavicular joint on the clavicle
		CSC	Centre of the sternoclavicular cartilage on the clavicle
Cartilage contours	Scapula	Cartilage1	Contour of the glenoid cartilage related to the glenohumeral joint
	Clavicle	Cartilage2	Contour of the clavicle cartilage related to the acromioclavicular joint

### 2.3 Bone coordinate systems

**FIGURE 2:**
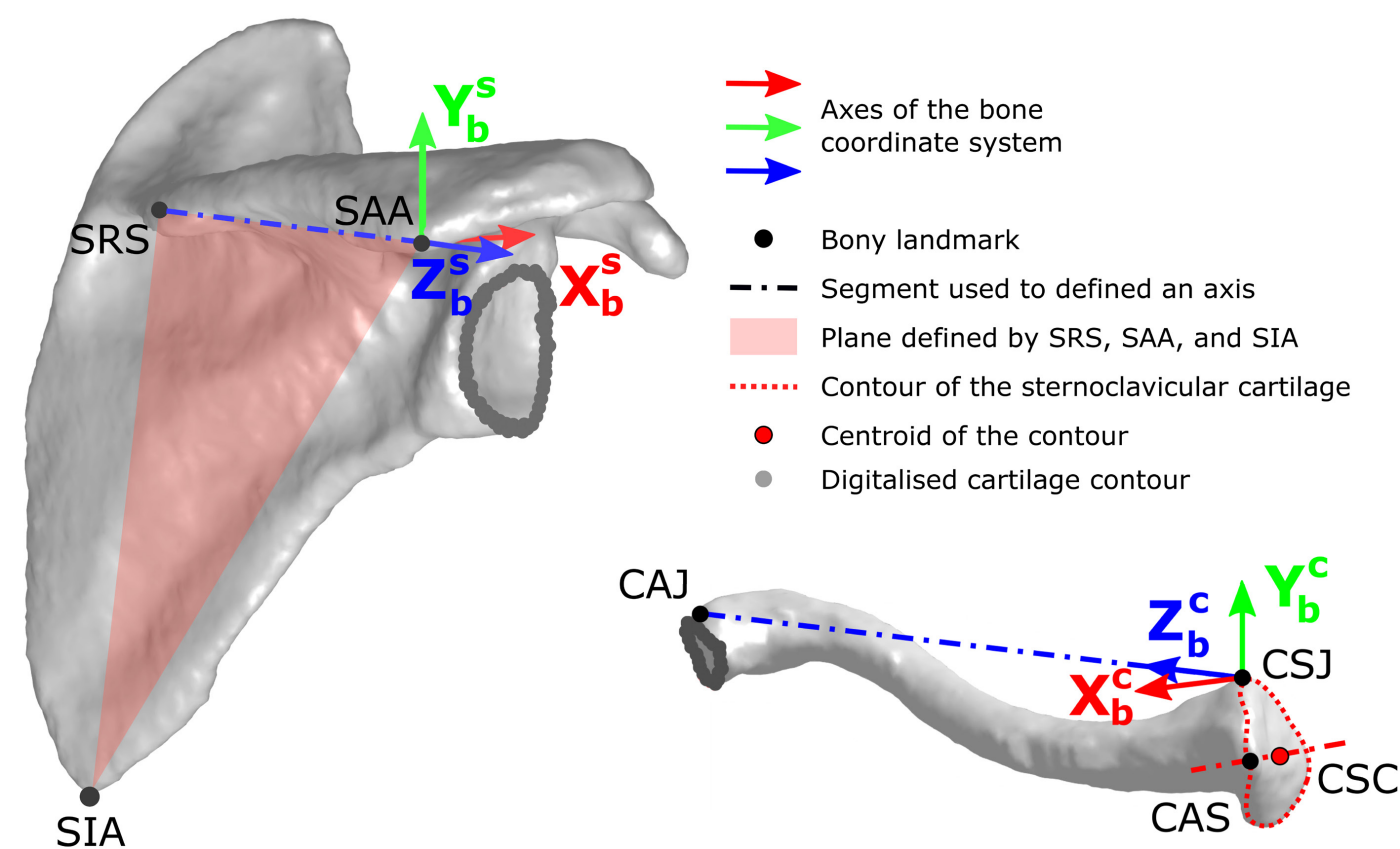
Illustration of the position and orientation of the digitalised cartilage contours. The position and orientation of digitalised cartilage contours are illustrated in relation to the related for a right scapula and clavicle. The geometrical elements used to define bone coordinate system axes are also reported: SAA: origin of the scapula coordinate system, $Z_{s}^{b}$: The line connecting SRS and SAA, pointing to SAA, $X_{s}^{b}$: The line perpendicular to the plane formed by SIA, SAA, and SRS, pointing forward. $Y_{s}^{b}$: The common line perpendicular to $X_{s}^{b}$ and $Z_{s}^{b}$, pointing upward; CSJ: origin of the clavicle coordinate system, $Z_{c}^{b}$: The line connecting CSJ and CAJ, pointing to CAJ, $X_{c}^{b}$: The line connecting CAS to CSC, i.e., the centre of the sternoclavicular cartilage, pointing forward, $Y_{c}^{b}$: The common line perpendicular to $X_{c}^{b}$ and $Z_{c}^{b}$, pointing upward.

To allow comparison between digitalisations, the 3D point clouds defining each cartilage contour were expressed in a bone coordinate system (orthonormal coordinate system: X_b_, Y_b_, Z_b_) (Figure 2). This coordinate system was defined using the previously defined anatomical landmarks following the recommendations of the International Society of Biomechanics (ISB) (Wu et al., 2005). Only $X_{c}^{b}$ of the clavicle was defined using another approach, as the thorax, needed in ISB recommendations, was not available is this study. For that, the centre of the sternoclavicular joint was used in combination with the anterior border apex of this joint to define the clavicle antero‐posterior axis. This procedure is similar to the one previously proposed by Gutierrez Delgado et al. (2017). To record the position and orientation of the bone coordinate system, each bone was also instrumented with a double dodecahedron made of ArUco markers (Figure 1).

### 2.4 Position, orientation, and shape of the cartilage contour

**FIGURE 3:**
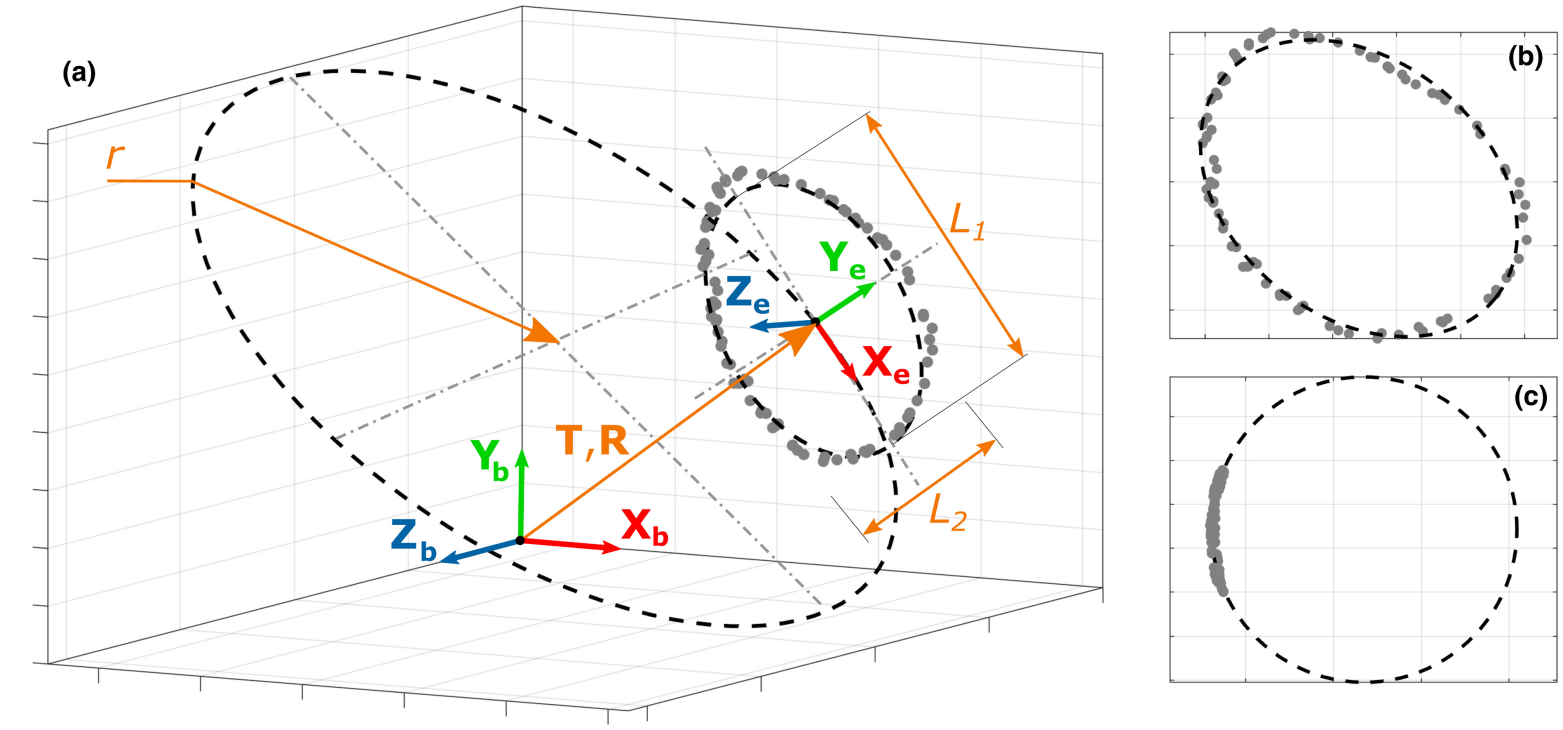
Parametrisation of a measured 3D point cloud defining a cartilage contour. (a) 3D point cloud (grey points), coordinate systems (bone coordinate system: $X_{b},Y_{b},Z_{b}$, ellipse coordinate system: $X_{e},Y_{e},Z_{e}$) and related parameters ($T:\;X_{\text{cart}},Y_{\text{cart}},Z_{\text{cart}}$ – 3D position of the ellipse in the bone coordinate system, $R:\;\alpha_{\text{cart}},\beta_{\text{cart}},\gamma_{\text{cart}}$ – 3D orientation of the ellipse in the bone coordinate system, L_1_ – length of the ellipse major axis, *L*_2_ – length of the ellipse minor axis, and *r* – radius of the least‐squares circle). (b) Orthogonal projection of the 3D point cloud on its least‐squares plane and fitting with an ellipse using a least‐squares approach based on orthogonal distance. (c) Orthogonal projection of the 3D point cloud on a plane having a normal vector along the minor axis of the previously defined ellipse and fitting with a least‐squares circle.

Firstly, the cartilage contour 3D point cloud (expressed in the related bone coordinate system) was projected orthogonally on its least‐squares plane (Figure 3a). The resulting 2D point cloud was fitted with an ellipse using a least‐squares approach based on orthogonal distance (Dingler, 2021) (Figure 3b). An ellipse coordinate system (orthonormal coordinate system: X_e_, Y_e_, Z_e_), centred on the resulting ellipse centre, was defined using three unit vectors: along the ellipse major axis, along the ellipse minor axis, and along the least‐squares plane normal vector pointing laterally (Figure 3a). Secondly, the cartilage 3D point cloud contour was projected orthogonally on a plane having as the normal vector the previously defined unit vector along the ellipse minor axis (Figure 3c). The resulting 2D point cloud was fitted with a least‐squares circle (Figure 3a).

A cartilage contour was thus defined by nine parameters. Six parameters were used to describe its 3D position ($T:\;X_{\text{cart}},Y_{\text{cart}},Z_{\text{cart}}$) and orientation ($R:\;\alpha_{\text{cart}},\beta_{\text{cart}},\gamma_{\text{cart}}$) in the bone coordinate system. Three other parameters were used to describe its shape: ellipse major and minor axis length (*L*_1_ and *L*_2_, i.e., length and width, respectively) and the least‐squares circle radius (*r*). Left scapulas were transformed as right scapulas to homogenise data.

### 2.5 Statistical analysis

Based on the previously established dataset, the reliability of the ArUco‐based localisation stylus to digitalise cartilage contours was assessed. Intra‐observer repeatability and inter‐observer reproducibility were computed using an ICC (Mokkink et al., 2016), respectively, ${\mathrm{ICC}}_{\text{intra}}$ and ${\mathrm{ICC}}_{\text{inter}}$. Intra‐observer repeatability represents in this study the degree of agreement between the results obtained by the same examiner across the three consecutive digitalisations of each cartilage contour. Inter‐observer repeatability represents the degree of agreement among the three examiners during digitalisation of each cartilage contour. A single measure, two‐way, mixed model‐type absolute ICC coefficients were used, that is, ICC(3,k) (Shrout & Fleiss, 1979). Variance components were estimated first with the *lme4* package (1.1–28) (R‐Core‐Team, 2020b). The total variance was defined as the class component variance sum:

(1) $$\sigma_{\text{total}}^{2}={\sigma_{\text{cartilage}}^{2}+\sigma }_{\text{operator}}^{2}+\sigma_{\text{digitalisation}}^{2}+\sigma_{\text{residual}}^{2}$$

where $\sigma_{\text{cartilage}}^{2}$, $\sigma_{\text{examiner}}^{2}$, $\sigma_{\text{digitalisation}}^{2}$, and $\sigma_{\text{residual}}^{2}$ are, respectively, the cartilage, the examiner, the digitalisation, and the residual variance. Following the methodology proposed by Chia and Sangeux (2017), ICC estimates were then obtained as follows:

(2) $$\begin{array}{c} {\mathrm{ICC}}_{\text{intra}}=\frac{\sigma_{\text{total}}^{2}-\left(\sigma_{\text{digitalisation}}^{2}+\sigma_{\text{residual}}^{2}\right)}{\sigma_{\text{total}}^{2}}\; \end{array}$$

(3) $$\begin{array}{c} {\mathrm{ICC}}_{\text{inter}}=\frac{\sigma_{\text{total}}^{2}-\left(\sigma_{\text{operator}}^{2}+\sigma_{\text{residual}}^{2}\right)}{\sigma_{\text{total}}^{2}}\; \end{array}$$

ICC estimates were qualified as relative reliability metrics and classified as poor (<0.5), moderate (0.5 to 0.75), good (0.75 to 0.90), and excellent (≥0.90) agreement (Koo & Li, 2016).

Standard error of measurement (SEM) was also quantified both for intra‐observer repeatability and inter‐observer reproducibility as an absolute reliability metric and computed as follows:

(4) $$\begin{array}{c} {\mathrm{SEM}}_{\text{intra}}=\sqrt{\sigma_{\text{total}}^{2}\times \left(1-{\mathrm{ICC}}_{\text{intra}}\right)}\; \end{array}$$

(5) $$\begin{array}{c} {\mathrm{SEM}}_{\text{inter}}=\sqrt{\sigma_{\text{total}}^{2}\times \left(1-{\mathrm{ICC}}_{\text{inter}}\right)}\; \end{array}$$

As each SEM value is related to a specific range and unit of measurement, %SEM was additionally calculated to aid interpretation. A classification criterion of acceptability was arbitrarily defined for each unit: excellent (<1 mm or °), good (1 mm or ° to 2 mm or °), moderate (2 mm or ° to 3 mm or °), and poor (3 mm or ° or higher).

The mean inter and intra‐observer 95% CI was computed as ±1.96 SEM (Mokkink et al., 2010). All computations related to the reliability analysis were performed in R 4.1.2 and RStudio (version 2021.09.0 build 351) (R‐Core‐Team, 2018).

## 3 RESULTS

Mean, standard deviation, confidence interval, and results of the statistical analysis are reported in Table 2 for the glenoid cartilage related to the glenohumeral joint and in Table 3 for the clavicle cartilage related to the acromioclavicular joint. Overall, 35.2 ± 2.4 s and 26.6 ± 10.2 s were required by each examiner to digitalise the contour of a glenoid cartilage and an acromioclavicular cartilage, respectively.

**TABLE 2:** Mean, standard deviation, confidence interval, ICC, and standard error of measurement observed for the glenoid cartilage related to the glenohumeral joint.

Parameter	Unit	Mean	Std	95% CI	Intra‐observer		Inter‐observer	
					ICC	SEM (SEM%)	ICC	SEM (SEM%)
$X_{\text{cart}}$	mm	31.8	3.1	31.1; 32.5	0.89 ^‡^	1.1 (3.6) ^‡^	0.85 ^‡^	1.3 (4.1) ^‡^
$Y_{\text{cart}}$	mm	−17.7	3.4	−18.4; −16.9	0.95 ^†^	1.0 (5.7) ^‡^	0.71 ^*^	2.3 (13.6) ^*^
$Z_{\text{cart}}$	mm	−16.6	6.6	−18.0; −15.1	1.00 ^†^	0.4 (2.4) ^†^	0.99 ^†^	0.6 (3.3) ^†^
$\alpha_{\text{cart}}$	deg	0.9	6.2	−0.5; 2.3	0.98 ^†^	0.9 (4.4) ^†^	0.97 ^†^	1.1 (5.3) ^‡^
$\beta_{\text{cart}}$	deg	10.4	3.4	9.6; 11.1	0.92 ^†^	1.1 (10.2) ^‡^	0.91 ^†^	1.2 (11.0) ^‡^
$\gamma_{\text{cart}}$	deg	−93.8	6.6	−95.3; −92.4	0.95 ^†^	1.7 (1.8) ^‡^	0.93 ^†^	1.9 (2.1) ^‡^
*L*_1_	mm	28.8	1.1	28.6; 29.0	0.92 ^†^	0.2 (1.1) ^†^	0.77 ^‡^	0.3 (1.9) ^†^
*L*_2_	mm	20.4	1.2	20.2; 20.7	0.96 ^†^	0.1 (1.1) ^†^	0.89 ^‡^	0.2 (1.8) ^†^
*r*	mm	38.1	5.5	36.9; 39.4	0.68 ^*^	3.0 (7.9) ^*^	0.64 ^*^	3.2 (8.4)

*Note*: **Parameters:** 3D position of the ellipse $X_{\text{cart}}$, $\gamma_{\text{cart}}$, $Z_{\text{cart}}$, 3D orientation of the ellipse $\alpha_{\text{cart}}$, $\beta_{\text{cart}}$, $\gamma_{\text{cart}}$, length of the ellipse major axis *L*_1_, length of the ellipse minor axis *L*_2_, and radius of the least‐squares circle *r*. **Classification:** ^†^excellent (in blue); ^‡^good (in green); *moderate (in orange); poor (black).

Abbreviations: CI 95%, 95% confidence interval; ICC, intra‐class correlation; SEM, standard error of measurement; Std, standard deviation.

**TABLE 3:** Mean, standard deviation, confidence interval, ICC, and standard error of measurement observed for the clavicle cartilage related to the acromioclavicular joint.

Parameter	Unit	Mean	Std	95% CI	Intra‐observer		Inter‐observer	
					ICC	SEM (SEM%)	ICC	SEM (SEM%)
$X_{\text{cart}}$	mm	0.1	2.7	−0.5; 0.6	0.90 ^†^	0.9 (9.0) ^†^	0.67 ^*^	1.6 (16.3) ^‡^
$Y_{\text{cart}}$	mm	−6.9	1.4	−7.2; −6.6	0.81 ^‡^	0.6 (8.8) ^†^	0.75 ^‡^	0.7 (10.2) ^†^
$Z_{\text{cart}}$	mm	134.9	7.9	133.3; 136.6	0.99 ^†^	0.7 (0.5) ^†^	0.97 ^†^	1.4 (1.0) ^‡^
$\alpha_{\text{cart}}$	deg	21.9	13.8	19.0; 24.8	0.98 ^†^	2.0 (6.2) ^‡^	0.94 ^†^	3.2 (10.0)
$\beta_{\text{cart}}$	deg	39.0	11.6	36.6; 41.4	0.99 ^†^	1.1 (2.9) ^‡^	0.98 ^†^	1.7 (4.4) ^‡^
$\gamma_{\text{cart}}$	deg	−6.0	12.5	−8.6; −3.3	0.81 ^‡^	4.9 (11.1)	0.81 ^‡^	4.9 (11.1)
*L*_1_	mm	20.4	3.2	19.7; 21.0	0.98 ^†^	0.3 (2.5) ^†^	0.93 ^†^	0.5 (4.5) ^†^
*L*_2_	mm	13.0	1.2	12.7; 13.2	0.92 ^†^	0.2 (2.6) ^†^	0.78 ^‡^	0.3 (4.3) ^†^
*r*	mm	18.3	9.9	16.2; 20.4	0.93 ^†^	2.8 (15.4) ^*^	0.73 ^*^	5.4 (29.6)

*Note*: **Parameters:** 3D position of the ellipse $X_{\text{cart}}$, $\gamma_{\text{cart}}$, $Z_{\text{cart}}$, 3D orientation of the ellipse $\alpha_{\text{cart}}$, $\beta_{\text{cart}}$, $\gamma_{\text{cart}}$, length of the ellipse major axis *L*_1_, length of the ellipse minor axis *L*_2_, and radius of the least‐squares circle *r*. **Classification:** ^†^excellent (in blue); ^‡^good (in green); *moderate (in orange).

Abbreviations: CI 95%, 95% confidence interval; ICC, intra‐class correlation; SEM, standard error of measurement; Std, standard deviation.

Concerning the glenoid cartilage (Table 2), the intra‐observer relative reproducibility always demonstrated good‐to‐excellent agreement, with the ICC ranging between 0.89 and 1.00, associated with good‐to‐excellent SEM values, except for the radius of the least‐squares circle (ICC = 0.68, SEM = 3.0 mm). For this parameter, the mean intra‐observer 95% CI of the mean was 5.9 mm. The inter‐observer relative reproducibility generally demonstrated good‐to‐excellent agreement, with the ICC ranging between 0.77 and 0.99, associated with good‐to‐excellent SEM values. Only the cartilage position along the inferior–superior $Y_{s}^{b}$ axis (ICC = 0.71, SEM = 2.3 mm) and the least‐squares circle radius (ICC = 0.64, SEM = 3.2 mm) demonstrated poorer results. For these two parameters, the mean intra‐observer 95% CI of the mean was 4.5 and 5.2 mm, respectively.

Concerning the clavicle cartilage (Table 3), the intra‐observer relative reproducibility always demonstrated good‐to‐excellent agreement, with the ICC ranging between 0.81 and 0.99. However, the cartilage orientation around the medial‐lateral $Z_{b}^{s}$ axis (ICC = 0.81, SEM = 4.9°) and the least‐squares circle radius (ICC = 0.93, SEM = 2.8 mm) demonstrated poorer absolute reproducibility results. The inter‐observer relative reproducibility generally demonstrated good‐to‐excellent agreement, with the ICC ranging between 0.75 and 0.98. The cartilage position along the antero‐posterior $X_{c}^{b}$ (ICC = 0.67, moderate agreement) and the least‐squares circle radius (ICC = 0.73, moderate agreement) demonstrated poorer results. For these two parameters, the mean intra‐observer 95% CI of the mean was 3.1 mm and 10.6 mm, respectively. Furthermore, the cartilage orientation around the anterior–posterior $X_{s}^{b}$ (ICC = 0.94, SEM = 3.2°) and the medial–lateral $Z_{s}^{b}$ (ICC = 0.81, SEM = 4.9°) axes demonstrated poorer absolute reproducibility results.

## 4 DISCUSSION

Various 3D scanning systems are available to digitalise anatomical structures (Govender et al., 2023; Sindhu & Soundarapandian, 2019). This study was focused on a manual tactile contact method based on a hand‐held stylus made of ArUco markers (Garrido‐Jurado et al., 2014; Wu et al., 2017). In particular, the reliability of this simple approach was assessed during the 3D digitalisation of shoulder cartilage contours.

The proposed parameters describing cartilage contours allow for shape analysis of these structures. Concerning the glenoid cartilage, its mean length and width, i.e., the articular surface of the glenoid fossa excluding the labrum, were, respectively, 28.8 ± 1.2 and 20.4 ± 1.2 mm. These dimensions are consistent with the mean glenoid cartilage shape measured by Hata et al. (1992) (length: 33.5 ± 4.1 mm, width: 26.5 ± 3.2 mm) on a larger population (*N* = 31 shoulders). The 3D radius of curvature was 38.1 ± 5.5 mm, which is also consistent with previous measurements. Indeed, McPherson et al. measured on 93 shoulders a mean glenoid radius of curvature of 32.2 ± 7.6 and 40.6 ± 14 mm in antero‐posterior and axillary–lateral views, respectively (Mcpherson et al., 1997). To the best of our knowledge, the glenoid cartilage position related to a scapula coordinate system is rarely reported in the literature. Furthermore, the glenoid cartilage orientation, in particular glenoid version and inclination, is still commonly measured in 2D based on radiographs (Mansat & Bonnevialle, 2013) and cannot be directly compared to the presents results expressed in the scapula ISB coordinate system. Concerning the acromioclavicular cartilage, its mean length and width were, respectively, 20.4 ± 3.2 and 13.0 ± 1.2 mm. These dimensions are consistent with the mean shape measured by Nakazawa et al. (2016) (length: 20.5 [17; 33] mm, width: 8.2 [5; 13] mm) on the external third of the clavicle bone using CT scan (*N* = 10 shoulders) (Nourissat et al., 2007). The 3D radius of curvature was 18.3 ± 9.9 mm. This was the shape parameter most affected by variability. This can be explained by the various morphologies of the acromioclavicular joint reported in the literature, defining various joint curvatures (Colegate‐Stone et al., 2010). As for the glenoid cartilage, comparison of the acromioclavicular cartilage position and orientation in a clavicle coordinate system is made difficult by the use of various coordinate systems or because of data scarcity.

The present results generally demonstrate a good‐to‐excellent inter and intra‐observer reliability both on medium‐size (glenoid cartilage) and small‐size (acromioclavicular cartilage) cartilage contours. This supports the fact that hand‐held stylus can be valuable tools for 3D digitalisation, as previously demonstrated on knee ligaments (Athwal et al., 2020). In particular, such a digitalisation is often performed after dissection, which can allow visual identification of surrounding anatomical structures. However, as being guided by the operator's vision, this approach can lead to several discrepancies when the targeted structure is not well‐recognisable. Hence, it appeared that the inferior–superior limits of the glenoid cartilage were more difficult to identify in a reliable way, as well as the antero‐posterior limits of the clavicle cartilage. Unsurprisingly, cartilage borders in these directions are more prone to identification error due to other surrounding structures. Indeed, a fibrocartilaginous transition area between the glenoid cartilage and the labrum fibrous tissue in the superior and inferior parts of the glenoid is frequently observed (Cooper et al., 1992). Similarly, the anterior and posterior parts of the clavicle distal end correspond to ligament insertions and sometimes to a degenerated acromioclavicular disk (Nakazawa et al., 2016). It must also be noted that, even with a relative reliability being good to excellent, acromioclavicular cartilage orientation around the anterior–posterior and the medial–lateral axes demonstrated poor absolute reliability, with SEM values at 3.2° and 4.9°, respectively. Depending on the application, these levels might limit the capacity of the approach to provide reliable orientations in small structures such as the acromioclavicular cartilage.

This study has some limitations. First, due to the mean age of the specimens (77.4 ± 9.99 years), cartilage morphologic changes may have appeared due to various progression of osteoarthritis (Pritzker et al., 2006) potentially affecting identification of cartilage surfaces. Still, the present results suggest that a good‐to‐excellent agreement can be obtained in such conditions. Second, as the digitalisation process presented in this study is guided by vision, the operator's knowledge and experience is directly related to the reported results. In this sense, the present results are not directly generalisable, justifying the choice for the use of a single measure, two‐way mixed model ICC(3,k). Still, as the three operators presented very different backgrounds (F.M. – biomechanical researcher, C.E.K – instrumentation engineer, and P.R. – intern in orthopaedic trauma surgery), the reported levels of reproducibility are encouraging, while further research will be required to demonstrate the clinical applicability. Third, following the ISB recommendations, the *x*‐axis of the clavicle should be defined perpendicular to the *z*‐axis of the clavicle and the *y*‐axis of the thorax (Wu et al., 2005). As the thorax was not available is this study, another approach has been used to define the clavicle coordinate system (Gutierrez Delgado et al., 2017). It results that a rotational offset around the clavicle medial–lateral axis may appear between the approach used in this study and ISB recommendations. Fourth, the parameter set used to describe the cartilage position, orientation, and shape might not be adaptable to every cartilage type. In particular, some cartilages are flatter than others (Emura et al., 2014), leading to discrepancies when trying to fit a sphere to estimate their curvature. This may explain the moderate reproducibility results obtained on the curvature *r* parameter for both analysed cartilages. This could be improved by defining a curvature threshold allowing to discriminate flat cartilages.

## 5 CONCLUSION

The use of an ArUco‐based localisation stylus can thus be a reliable and accurate (Le et al., 2022) approach to digitalise anatomical structures such as shoulder cartilage contours. Furthermore, it represents a fast and cost‐effective approach in constrast to the current gold standard, that is, MRI (Hayashi et al., 2011), known as being costly and time‐consuming. Still, further research is required to compare the two approaches.

## AUTHOR CONTRIBUTIONS

**Nicolas Holzer and Jean‐Yves Beaulieu:** Supervision. **Pierre Vacher and Nicolas Holzer:** Funding acquisition. **Florent Moissenet, Christian Elmo Kulanesan, and Pablo Rodriguez:** Conceptualisation. **Florent Moissenet, Christian Elmo Kulanesan, Pablo Rodriguez, and Jean‐Yves Beaulieu:** Methodology. **Florent Moissenet, Christian Elmo Kulanesan, and Pablo Rodriguez:** Data collection. **Florent Moissenet and Christian Elmo Kulanesan:** Data curation. **Florent Moissenet, Christian Elmo Kulanesan, and Kevin Co:** Data processing. **Florent Moissenet:** Writing—original draft. **Florent Moissenet, Christian Elmo Kulanesan, Kevin Co, Pablo Rodriguez, Pierre Vacher, Jean‐Yves Beaulieu, and Nicolas Holzer:** Writing—review & editing. All Authors have read and agreed to the published version of the manuscript.

## CONFLICT OF INTEREST STATEMENT

The authors certify that they have no affiliations with or involvement in any organisation or entity with any financial interest or non‐financial interest in the subject matter or materials discussed in this manuscript.

## Data Availability

Raw and processed cameras images as well as Python code used to process images are available on the GitHub repository (https://github.com/elmokulc/roboshoulder).
